# Multidimensional Sleep Health Prior to SARS-CoV-2 Infection and Risk of Post–COVID-19 Condition

**DOI:** 10.1001/jamanetworkopen.2023.15885

**Published:** 2023-05-30

**Authors:** Siwen Wang, Tianyi Huang, Marc G. Weisskopf, Jae H. Kang, Jorge E. Chavarro, Andrea L. Roberts

**Affiliations:** 1 Department of Nutrition, Harvard T. H. Chan School of Public Health, Boston, Massachusetts; 2 Channing Division of Network Medicine, Department of Medicine, Brigham and Women’s Hospital and Harvard Medical School, Boston, Massachusetts; 3 Department of Environmental Health, Harvard T. H. Chan School of Public Health, Boston, Massachusetts; 4 Department of Epidemiology, Harvard T. H. Chan School of Public Health, Boston, Massachusetts

## Abstract

**Importance:**

The association of multiple healthy sleep dimensions with post–COVID-19 condition (PCC), also known as long COVID, has not been investigated.

**Objective:**

To examine whether multidimensional sleep health before and during the COVID-19 pandemic, prior to SARS-CoV-2 infection, was associated with the risk of PCC.

**Design, Setting, and Participants:**

This prospective cohort study (2015-2021) included Nurses’ Health Study II participants who reported testing positive (n = 2303) for SARS-CoV-2 infection in a substudy series of COVID-19–related surveys (n = 32 249) between April 2020 and November 2021. After exclusion for incomplete information about sleep health and nonresponse to a question about PCC, 1979 women were included in the analysis.

**Exposures:**

Sleep health was measured both before (June 1, 2015, to May 31, 2017) and early (April 1 to August 31, 2020) in the COVID-19 pandemic. Prepandemic sleep score was defined according to 5 dimensions: morning chronotype (assessed in 2015), 7 to 8 hours of sleep per day, low insomnia symptoms, no snoring, and no frequent daytime dysfunction (all assessed in 2017). On the first COVID-19 substudy survey (returned between April and August 2020), average daily sleep duration and sleep quality for the past 7 days were queried.

**Main Outcomes and Measures:**

SARS-CoV-2 infection and PCC (≥4 weeks of symptoms) were self-reported during 1 year of follow-up. Comparisons were examined between June 8, 2022, and January 9, 2023, using Poisson regression models.

**Results:**

Of the 1979 participants reporting SARS-CoV-2 infection (mean [SD] age, 64.7 [4.6] years; 1979 [100%] female; and 1924 [97.2%] White vs 55 [2.8%] other races and ethnicities), 845 (42.7%) were frontline health care workers, and 870 (44.0%) developed PCC. Compared with women who had a prepandemic sleep score of 0 or 1 (least healthy), those who scored 5 (most healthy) had a 30% lower risk of developing PCC (multivariable-adjusted relative risk, 0.70; 95% CI, 0.52-0.94; *P* for trend <.001). Associations did not differ by health care worker status. No or little daytime dysfunction prepandemic and good sleep quality during the pandemic were independently associated with a lower risk of PCC (relative risk, 0.83 [95% CI, 0.71-0.98] and 0.82 [95% CI, 0.69-0.99], respectively). Results were similar when PCC was defined as having 8 or more weeks of symptoms or as having ongoing symptoms at the time of PCC assessment.

**Conclusions and Relevance:**

The findings indicate that healthy sleep measured prior to SARS-CoV-2 infection, both before and during the COVID-19 pandemic, may be protective against PCC. Future research should investigate whether interventions on sleep health may prevent PCC or improve PCC symptoms.

## Key Points

**Question:**

Is healthy sleep, both before and during the COVID-19 pandemic, prior to SARS-CoV-2 infection, protective against post–COVID-19 condition (PCC)?

**Findings:**

In this cohort study of 1979 women who reported testing positive for SARS-CoV-2, adherence to healthy sleep before infection was inversely associated with the risk of PCC. Compared with women who had a prepandemic sleep score of 0 or 1 (least healthy), those who scored 5 (most healthy) had a 30% lower risk of PCC.

**Meaning:**

These findings suggest that preinfection healthy sleep may be associated with a substantially decreased risk of PCC, indicating a need for further research on sleep health to prevent or improve symptoms of PCC.

## Teaser

This cohort study uses data from the Nurses’ Health Study II to examine the association of multidimensional sleep health with post–COVID-19 condition.

## Introduction

Post–COVID-19 condition (PCC), also known as long COVID, defined as having persistent COVID-19–related symptoms more than 4 weeks after infection,^1^ affects 20% to 70% of persons infected with SARS-CoV-2.^2,3,4,5,6,7,8^ Post–COVID-19 condition encompasses a wide range of symptoms that may impair daily functioning, including persistent cough, depression, fatigue, anosmia, and brain fog.^9,10^ Notwithstanding the serious public health burden caused by PCC, the etiology, prevention, and treatment of PCC are largely unknown.

Sleep problems are prevalent yet commonly overlooked health risk factors, affecting approximately 1 in 3 people worldwide.^11,12^ Sleep deprivation, poor sleep quality, and sleep disorders may lead to adverse physical, cognitive, and psychiatric consequences.^12^ The COVID-19 pandemic compounded both the severity of preexisting sleep disorders and the high prevalence of sleep disturbances.^13,14^ Unhealthy sleep dimensions individually and in combination (eg, late chronotype, short or long duration of sleep, snoring and sleep apnea, and daytime sleepiness) are prospectively associated with COVID-19 susceptibility, severity, and mortality.^15,16,17,18^ Mendelian randomization analyses have further identified insomnia, extreme sleep duration, and excessive daytime sleepiness as causal risk factors for the severity of COVID-19 and other respiratory infections.^16,17,19^ These unhealthy sleep dimensions have been linked to chronic low-grade inflammation and immune abberations,^12,20,21^ which have been implicated in the pathogenesis of PCC and other postinfection syndromes, such as postviral fatigue syndrome.^22,23,24^ Despite experimental evidence supporting the role of sleep deprivation and disturbances in infectious disease prognosis via immune dysfunction,^20,25^ the association between sleep health prior to SARS-CoV-2 infection and risk of PCC has not been investigated.

Leveraging data from a large longitudinal study, the Nurses’ Health Study II (NHS II), we prospectively evaluated the association of multidimensional sleep health prior to infection, measured before and early in the pandemic, with the risk of developing PCC among participants subsequently infected with SARS-CoV-2. We hypothesized that healthier sleep is associated with a lower risk of PCC.

## Methods

### Study Design and Population

In this cohort study, participants were drawn from the NHS II, which was established in 1989 when 116 429 US female nurses (aged 25-42 years) were enrolled.^26^ Health-related information was updated biennially via questionnaire. Among active NHS II participants, a web-based COVID-19 substudy was launched in April 2020 (henceforth termed baseline). Nurses’ Health Study II participants for whom we had an email address, who had returned the most recent main cohort questionnaire, and who were not currently participating in another substudy were invited (55 925 invited, 39 137 responded [71%]). The follow-up was monthly and quarterly. The final substudy follow-up questionnaire (henceforth termed final questionnaire) was administered 12 months after baseline. The end of follow-up for our analyses was November 3, 2021 (eMethods in Supplement 1).

Among 32 249 substudy participants who returned both the baseline and final questionnaires, 2303 reported a positive SARS-CoV-2 test during the 19 months of follow-up. Of these participants, we excluded 91 who did not have complete information about sleep health and 233 who did not respond to a question about PCC, leaving 1979 in the analysis (eFigure 1 in Supplement 1). The study was approved by the Brigham and Women’s Hospital institutional review board. Return of questionnaires constituted implied consent. This study followed the Strengthening the Reporting of Observational Studies in Epidemiology (STROBE) reporting guideline for cohort studies.

### Assessment of Sleep Dimensions

We measured sleep health before the pandemic (5 dimensions; June 1, 2015, to May 31, 2017, from which we created a healthy sleep score) and during the pandemic (2 dimensions; April 1 to August 31, 2020). Five prepandemic sleep dimensions were queried in 2015 (chronotype) and 2017 (sleep duration, insomnia, snoring, and daytime dysfunction). To assess chronotype, participants were asked to classify themselves as morning or evening people (“One hears about morning and evening types of people. Which one of these types do you consider yourself to be: definite morning, more of a morning, more of an evening, definite evening, or neither?”). This measure has been validated against the criterion standard human circadian marker, dim light melatonin onset.^27,28^ Daily sleep duration was the average number of sleep hours over a 24-hour period. Spearman correlation between self-reported sleep duration and a sleep diary was 0.79 (*P* < .001).^29^ Insomnia was assessed using a validated insomnia rating scale. Participants were asked how frequently they (1) have trouble falling asleep, (2) wake up several times at night, (3) wake up earlier than planned, and (4) have trouble getting back to sleep after waking too early.^30^ For each question, response options ranged from no (score 0) to 5 or more times per week (score 4). Items were summed to form an insomnia score (range, 0-16), where a score of less than 9 was considered low insomnia symptoms.^31^ For snoring, participants were asked, “How often do you snore?” Self-report of snoring had high sensitivity (90%) in detecting sleep apnea.^32^ Regarding daytime dysfunction, participants were asked to what extent daily function (eg, fatigue, mood, ability to work, concentration, memory) was affected by not being well-rested, with options from not at all to very much. This question has been used as a proxy for sleep quality.^33^

We calculated a prepandemic (2015-2017) sleep score: morning chronotype (definite morning or more of a morning person), adequate sleep (7-8 hours per day), low insomnia symptoms (0-8), no self-reported snoring, and no or little daytime dysfunction. Participants received a score of 1 if they met the criterion and 0 otherwise. For each participant, we calculated the total score by summing healthy sleep dimensions (0-5; higher score indicates healthier sleep). Because only 33 participants scored 0, we combined 0 and 1 for the analysis. A similar sleep score has been associated with health benefits, including lower risk of heart disease and longevity.^21,34,35,36^

At COVID-19 substudy baseline, participants were asked about their average daily sleep duration (in hours) and sleep quality (from very bad to very good) in the past 7 days. To jointly capture sleep health both before (2015-2017) and early in the pandemic (April to August 2020), we created a variable in 6 levels. Prepandemic sleep was categorized as either unhealthy (score of 0-3) or healthy (score of 4-5),^21^ and during-pandemic sleep was a count of healthy sleep duration (7-8 hours per day vs other) and healthy sleep quality (fairly good or very good vs other), which ranged from a score of 0 to 2.

### SARS-CoV-2 Infection, Hospitalization, and PCC

Participants reported SARS-CoV-2 infection (confirmed by polymerase chain reaction, antigen, or antibody test), date of test, and hospitalization due to COVID-19 since return of the last questionnaire or, at baseline, since March 1, 2020, on each substudy questionnaire. During the study period, there were 5 major peaks in the US, with dominant variants being Alpha and Delta.^37^

Ascertainment of PCC in this cohort has been described.^38^ Briefly, on the final COVID-19 substudy questionnaires, participants were asked whether they had COVID-19–related symptoms lasting for more than 4 weeks; anyone who endorsed this question was considered to have PCC. Of those with PCC, we asked (1) which symptoms they had experienced (fatigue; shortness of breath or difficulty breathing; persistent cough; muscle, joint, or chest pain; smell or taste problems; confusion, disorientation, or brain fog; memory issues; depression, anxiety, or changes in mood; headache; intermittent fever; heart palpitations; rash, blisters, or welts; mouth or tongue ulcers; and other), (2) whether symptoms were ongoing, and (3) duration of symptoms.

### Covariates

Covariates were selected a priori as potential risk factors for PCC. Date of birth, race and ethnicity (American Indian or Alaska Native, Asian, Black or African American, Native Hawaiian or Pacific Islander, or White), and height were self-reported at cohort enrollment. We included race and ethnicity as a study variable because of the known racial and ethnic disparity in sleep health and PCC.^39,40^ Diet, including alcohol consumption, was measured in 2015 using a validated semiquantitative food frequency questionnaire.^41,42^ We adapted the Alternate Healthy Eating Index-2010 score to describe diet quality. Weight, physical activity, smoking history, subjective complaints in memory and cognition,^43,44^ and lifetime history of physician-diagnosed diseases (cancer, diabetes, hypertension, high cholesterol, and asthma) were assessed in 2017. Body mass index (BMI) was calculated as weight in kilograms divided by height in meters squared. Frontline health care worker status (defined as physically working at a site delivering care or not) was self-reported at baseline. Depression and anxiety symptoms at baseline were measured using the Patient Health Questionnaire-2 and the Generalized Anxiety Disorder 2-item, respectively.^45^ Date of COVID-19 vaccination was self-reported on quarterly follow-up surveys.

### Statistical Analysis

We first compared the prevalence of demographic and lifestyle factors according to missingness of exposure and outcome. We then calculated the correlation (ϕ-coefficient) between dimensions of sleep health. Missingness for each covariate was less than 3%. Indicator variables were used for any missing covariate for categorical variables; the median was imputed for continuous variables.^46^

Poisson regression models were used to estimate the relative risks (RRs) and 95% CIs for the associations of individual sleep dimensions (both as categorical and dichotomized variables) and sleep score (0-5) with risk of PCC, with adjustment for age (continuous, years), race and ethnicity (White vs American Indian or Alaska Native, Asian, Black or African American, or Native Hawaiian or Pacific Islander), frontline health care worker status (yes, no), lifestyle factors (BMI [continuous], physical activity [continuous, metabolic equivalent task-hours per day], smoking [never, past, or current], diet [quintiles, Alternate Healthy Index-2010], and alcohol consumption [0, 0.1-4.9, 5.0-14.9, 15.0-29.9, or ≥30.0 g per day]), and history of comorbidities (cancer, diabetes, hypertension, high cholesterol, and asthma [yes, no]). Because sleep dimensions are correlated with each other,^21^ we included a model mutually adjusted for all prepandemic healthy sleep dimensions to explore their independent associations with PCC. The linear trend test was performed by treating indicator levels as a continuous variable. Test for trend for sleep duration was not performed because of the well-established U-shaped relationship between sleep duration and health (with 7-9 hours per day being the healthiest level).^47^

Because frontline health care workers have a higher risk of infection and potentially different sleep behavior due to their job characteristics,^48^ we investigated whether associations differed by frontline health care worker status by adding a cross-product term in the model. The significance of multiplicative interaction was tested using the Wald test.

We also examined the joint association of sleep health before (2015-2017) and early (April to August 2020) in the pandemic with subsequent risk of PCC. To reduce risk of reverse causation, we excluded 174 participants who reported a positive SARS-CoV-2 test on the baseline questionnaire when pandemic sleep dimensions were assessed.

We conducted several sets of sensitivity analyses. First, alternative definitions of PCC were used. Post–COVID-19 condition was defined as having symptoms longer than 8 weeks and, to reduce risk of recall bias, as having ongoing symptoms at the time of PCC assessment. Because symptoms of sleep deprivation and PCC may overlap, we excluded from PCC cases 155 participants who reported only fatigue, headache, brain fog, memory issues, and depression as their symptoms. To account for the possibility of lack of testing, 912 participants with presumed SARS-CoV-2 infection (without a confirmatory test) were included in the analytic sample. Because vaccination may reduce the risk of PCC,^5^ we excluded 129 participants who had been vaccinated before infection. To account for preexisting PCC-like symptoms, we additionally adjusted for memory and cognitive issues (the summed score of 6 subjective memory and cognitive complaints) assessed in 2017.^43^ To explore whether the associations were similar among participants who had less severe acute phase illness, we restricted analysis to 1877 persons who were not hospitalized due to COVID-19. Because sleep disorders have been linked with an increased risk of mental health conditions and mental health has been associated with the risk of PCC,^38^ we additionally excluded 336 persons with probable anxiety and depression, as measured by the Patient Health Questionnaire-2 and Generalized Anxiety Disorder 2-item, at baseline.^45^ Finally, multiple imputation was used for missing data.^49^ All analyses were conducted between June 8, 2022, and January 9, 2023, using SAS, version 9.4 software (SAS Institute Inc). A 2-sided *P* < .05 was considered statistically significant.

## Results

The analytic sample comprised 1979 women (American Indian or Alaska Native, 9 [0.5%]; Asian, 22 [1.1%]; Black or African American, 22 [1.1%]; Native Hawaiian or Pacific Islander, 2 [0.1%]; and White, 1924 [97.2%]), of whom 845 (42.7%) were frontline health care workers. The mean (SD) age of the cohort was 64.7 (4.6) years (range, 55–75 years). Participants with complete exposure and outcome information (n = 1979) vs without (n = 324) were more likely to be White and frontline health care workers (eTable 1 in Supplement 1). At baseline, participants who had a healthier prepandemic (2015-2017) sleep score were more likely to be older and White, have a lower BMI, and adhere to a healthier lifestyle (high diet quality score, high physical activity level, and never smoking) and less likely to have type 2 diabetes, hypertension, and asthma (Table 1). Compared with non–frontline health care workers, frontline health care workers were younger and less likely to have comorbidities (eTable 2 in Supplement 1). Sleep dimensions were weakly to moderately correlated with each other (ϕ-coefficient range, −0.02 to 0.32) (eFigure 2 in Supplement 1).

**Table 1.** Age-Standardized Participant Characteristics by Sleep Score Before the COVID-19 Pandemic (2015-2017)

	Sleep score, No. (%)					
	0 or 1 (Worst sleep)	2	3	4	5 (Best sleep)
No. of participants (%)	166 (8.4)	345 (17.4)	617 (31.2)	613 (31.0)	238 (12.0)
Age, mean (SD), y^a^	64.2 (4.5)	64.6 (4.5)	64.5 (4.6)	64.8 (4.7)	65.4 (4.7)
Race and ethnicity					
American Indian or Alaska Native	0	2 (0.6)	3 (0.5)	3 (0.5)	1 (0.4)
Asian	4 (2.4)	6 (1.7)	6 (1.0)	3 (0.5)	3 (1.3)
Black or African American	0	4 (1.2)	10 (1.6)	7 (1.1)	1 (0.4)
Native Hawaiian or Pacific Islander	0	0	1 (0.2)	1 (0.2)	0
White	162 (97.5)	333 (96.6)	597 (96.8)	599 (97.7)	233 (97.9)
BMI, mean (SD)	30.1 (6.8)	29.3 (6.6)	28.8 (6.5)	27.4 (6.2)	27.5 (6.3)
AHEI-2010, mean (SD)	57.4 (12.6)	58.9 (12.0)	59.2 (11.6)	63.0 (11.8)	64.4 (11.8)
Alcohol, mean (SD), g/d	7.3 (12.2)	6.8 (10.1)	6.8 (11.4)	8.2 (11.9)	6.4 (11.3)
Physical activity, MET-h/wk	19 (22.9)	26.1 (37.5)	24.1 (28.3)	29.8 (29.5)	31.8 (33)
Smoking					
Never	111 (66.7)	224 (65.0)	399 (64.7)	401 (65.5)	166 (69.8)
Past	50 (30.4)	114 (33.0)	206 (33.4)	201 (32.7)	67 (28.1)
Current	5 (3.0)	7 (1.9)	12 (1.9)	11 (1.8)	5 (2.1)
Active health care worker	66 (39.5)	143 (41.6)	272 (44.0)	279 (45.5)	85 (35.6)
Depression symptoms, mean (SD)^b^	1.6 (1.6)	1.1 (1.4)	1.0 (1.3)	0.8 (1.2)	0.6 (1)
Anxiety symptoms, mean (SD)^c^	1.5 (1.5)	1.3 (1.5)	1.1 (1.4)	1.0 (1.3)	0.9 (1.2)
High cholesterol	114 (68.8)	206 (59.8)	408 (66.0)	345 (56.3)	137 (57.6)
Diabetes	29 (17.3)	58 (16.9)	75 (12.1)	53 (8.6)	23 (9.8)
Hypertension	99 (59.4)	157 (45.4)	260 (42.2)	240 (39.2)	92 (38.7)
Asthma	43 (26.0)	92 (26.6)	155 (25.1)	116 (18.9)	41 (17.4)
Cancer	37 (22.2)	75 (21.8)	127 (20.5)	120 (19.6)	56 (23.4)
Hospitalization due to COVID-19	10 (6.3)	22 (6.5)	30 (4.8)	31 (5.0)	9 (3.8)
Vaccinated (first dose) against COVID-19 at the time of infection	8 (4.8)	29 (8.5)	36 (5.9)	42 (6.9)	13 (5.5)
Healthy sleep score dimensions					
Morning chronotype	32 (19.1)	143 (41.3)	327 (53.0)	471 (76.8)	238 (100)
Sleep 7-8 h/d	22 (13.1)	108 (31.2)	354 (57.3)	523 (85.3)	238 (100)
Low insomnia symptoms	16 (9.4)	128 (37.1)	397 (64.4)	544 (88.7)	238 (100)
No self-reported snoring	25 (15.0)	85 (24.5)	242 (39.2)	328 (53.5)	238 (100)
Not at all or a little daytime dysfunction	39 (23.3)	227 (65.9)	531 (86.1)	586 (95.6)	238 (100)

Abbreviations: AHEI-2010, Alternate Healthy Eating Index-2010; BMI, body mass index (measured as weight in kilograms divided by height in meters squared); MET, metabolic equivalent task.

^a^ Not age standardized.

^b^ Measured at COVID substudy baseline (April to August 2020) using the Patient Health Questionnaire-2.

^c^ Measured at COVID substudy baseline (April to August 2020) using the Generalized Anxiety Disorder 2-item.

**Figure 1.**
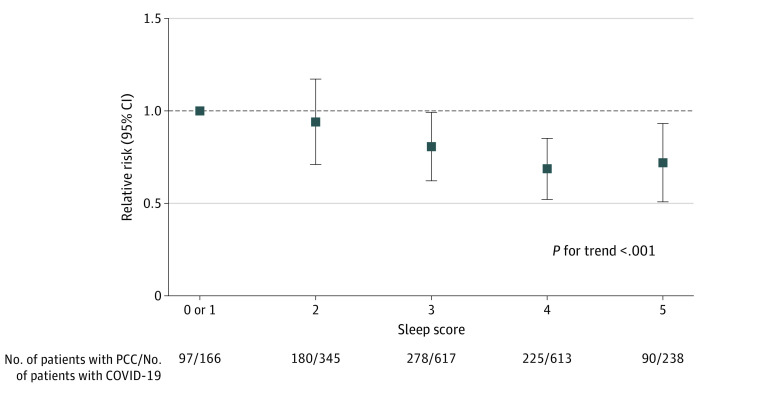
Sleep Score Before the COVID-19 Pandemic (2015-2017) and Risk of Post–COVID-19 Condition (PCC) Sleep score (0-5) included morning chronotype, 7 to 8 hours of sleep per day, low insomnia symptoms, no self-reported snoring, and not at all or a little daytime dysfunction. A higher score indicates better sleep. Adjusted for age, race and ethnicity, frontline health care worker status, smoking history, body mass index, healthy eating, alcohol intake, physical activity, and history of comorbidities. The linear trend test was performed by treating healthy sleep score as a continuous variable.

A total of 870 participants (44.0%) reported having PCC symptoms. Healthier prepandemic sleep score showed a dose-response association with lower risk of PCC (Figure 1). Compared with participants who had a sleep score of 0 or 1, those who scored 5 had a 30% lower risk of developing PCC (RR, 0.70; 95% CI, 0.52-0.94; *P* for trend <.001). The association between sleep score and PCC did not differ by health care worker status (eTable 3 in Supplement 1).

**Figure 2.**
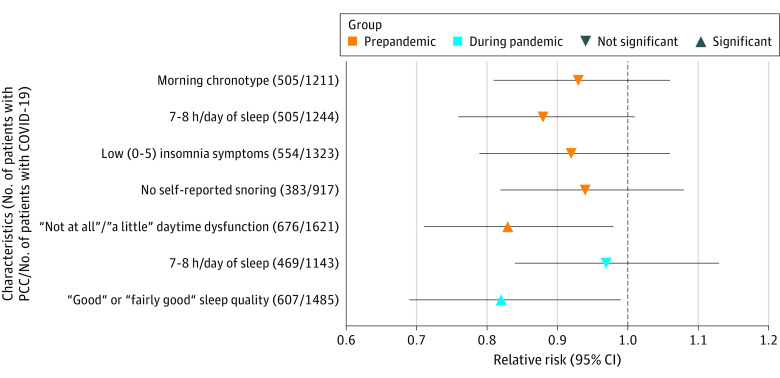
Associations of Individual Sleep Dimensions (Dichotomized) With Risk of Post–COVID-19 Condition (PCC) Models were adjusted for age, race and ethnicity, frontline health care worker status, smoking history, body mass index, healthy eating index score, alcohol intake, physical activity, and history of comorbidities and mutually adjusted for other healthy sleep dimensions measured at the same time point (ie, before [2015-2017] or during [April to August 2020] the pandemic). Morning chronotype included more of a morning person and definitely a morning person. Participants who at baseline reported testing positive for SARS-CoV-2 were excluded from analyses to reduce risk of reverse causation (n = 174).

Of the 5 sleep dimensions assessed prior to the pandemic (2015-2017), chronotype, sleep duration, insomnia, and daytime dysfunction were associated with risk of PCC after adjusting for age and race and ethnicity (Table 2, model 1). Sleep quality early in the pandemic was associated with risk of PCC (Table 2, model 1). Sleep durations of 6, 7, 8, 9, and 10 or more hours were not significantly associated with a lower risk of PCC vs 5 hours or less. The strongest associations were found for daytime dysfunction before the pandemic and sleep quality during the pandemic. Associations were attenuated by 0% to 13% after further adjustment for health risk factors (Table 2, model 2). Sleep duration, insomnia, and daytime dysfunction prior to the pandemic and sleep quality early in the pandemic remained significantly associated with risk of PCC. Similar associations were observed when each sleep dimension was evaluated as a binary variable (eTable 4 in Supplement 1; Figure 2). When we included all prepandemic sleep dimensions in the same model, only daytime dysfunction was independently associated with a lower risk of PCC (RR, 0.83; 95% CI, 0.71-0.98) (Figure 2). Similarly, sleep quality during the pandemic was associated with lower risk of PCC (RR, 0.82; 95% CI, 0.69-0.99) In analyses jointly examining sleep dimensions prior to and early in the pandemic, participants who had healthy sleep at both time points were at lowest risk of PCC (RR, 0.64; 95% CI, 0.50-0.82) vs those who had a poor to intermediate sleep score prior to the pandemic and had neither optimal sleep duration (ie, 7 hours per day) nor fairly good or good sleep quality early in the pandemic (Table 3).

**Table 2.** Associations of Individual Sleep Dimensions Before (2015-2017) and Early (April to August 2020) in the COVID-19 Pandemic With Risk of PCC

Healthy sleep dimension	No. with PCC/total No. with COVID-19 (%)	RR (95% CI)	
		Model 1, adjusted for age and race and ethnicity	Model 2, additionally adjusted for health-related factors^a^
**Before the pandemic**			
Chronotype			
Definitely a morning person	276/662 (41.7)	0.79 (0.64-0.99)	0.84 (0.67-1.05)
More of a morning person	229/549 (41.7)	0.80 (0.64-0.99)	0.84 (0.67-1.05)
More of an evening person	208/462 (45.0)	0.86 (0.68-1.08)	0.87 (0.69-1.09)
Definitely an evening person	117/223 (52.5)	1 [Reference]	1 [Reference]
Neither	40/83 (48.2)	0.92 (0.64-1.32)	0.97 (0.68-1.39)
*P* value for trend^b^	NA	.048	.18
Sleep duration, h/d			
≤5	77/150 (51.3)	1 [Reference]	1 [Reference]
6	242/478 (50.6)	0.98 (0.76-1.27)	1.00 (0.77-1.30)
7	327/796 (41.1)	0.80 (0.62-1.02)	0.84 (0.65-1.08)
8	178/448 (39.7)	0.77 (0.59-1.01)	0.80 (0.61-1.05)
9	37/88 (42.1)	0.81 (0.55-1.20)	0.79 (0.53-1.18)
≥10	9/19 (47.4)	0.91 (0.46-1.83)	0.95 (0.47-1.91)
Insomnia symptoms, points			
0-2	117/310 (37.7)	0.78 (0.63-0.97)	0.79 (0.64-0.98)
3-5	202/504 (40.1)	0.83 (0.70-0.99)	0.83 (0.69-0.99)
6-8	235/509 (46.2)	0.96 (0.81-1.13)	0.95 (0.80-1.13)
≥9	316/656 (48.2)	1 [Reference]	1 [Reference]
*P* value for trend	NA	.007	.009
Self-reported snoring			
Every night	112/222 (50.5)	1 [Reference]	1 [Reference]
Most nights	112/262 (42.8)	0.85 (0.65-1.10)	0.90 (0.69-1.17)
A few nights/wk	83/177 (46.9)	0.93 (0.70-1.23)	1.02 (0.77-1.37)
Occasionally	180/401 (44.9)	0.89 (0.70-1.13)	1.00 (0.79-1.28)
Almost never	201/482 (41.7)	0.83 (0.66-1.04)	0.94 (0.74-1.19)
Do not know	182/435 (41.8)	0.83 (0.65-1.05)	0.90 (0.71-1.15)
*P* value for trend^c^	NA	.21	.87
Daytime dysfunction			
Not at all	238/656 (36.3)	0.54 (0.33-0.87)	0.55 (0.34-0.89)
A little	438/965 (45.4)	0.68 (0.42-1.09)	0.66 (0.41-1.07)
Moderate	119/237 (50.2)	0.75 (0.46-1.23)	0.71 (0.43-1.17)
Quite a bit	57/94 (60.6)	0.91 (0.53-1.54)	0.89 (0.52-1.51)
Very much	18/27 (66.7)	1 [Reference]	1 [Reference]
*P* value for trend	NA	<.001	<.001
**Early in the pandemic** ^d^			
Sleep duration, h/d			
≤5	54/106 (50.9)	1 [Reference]	1 [Reference]
6	150/316 (47.5)	0.93 (0.68-1.27)	0.96 (0.70-1.32)
7	265/624 (42.5)	0.83 (0.62-1.12)	0.88 (0.65-1.18)
8	204/419 (39.3)	0.77 (0.57-1.04)	0.81 (0.60-1.10)
9	68/175 (38.9)	0.76 (0.53-1.09)	0.77 (0.54-1.10)
≥10	31/64 (48.4)	0.94 (0.60-1.47)	0.92 (0.59-1.43)
Sleep quality			
Very good	116/331 (35.1)	0.53 (0.31-0.91)	0.57 (0.33-0.98)
Fairly good	491/1154 (42.6)	0.65 (0.39-1.09)	0.69 (0.41-1.15)
Fairly bad	150/296 (50.7)	0.78 (0.46-1.32)	0.80 (0.46-1.36)
Very bad	15/23 (65.2)	1 [Reference]	1 [Reference]
*P* value for trend	NA	<.001	<.001

Abbreviations: NA, not applicable; PCC, post–COVID-19 condition; RR, relative risk.

^a^ Model 1 plus frontline health care worker status, smoking history, body mass index, Alternate Healthy Eating Index-2010 score, alcohol intake, physical activity, and history of comorbidities. Sleep dimensions were not mutually adjusted. The linear trend test was performed by treating healthy sleep score as a continuous variable. Linear trend for duration of sleep was not calculated due to the known U-shaped relationship between sleep duration and health outcomes.

^b^ Excluded “neither” from the trend analysis.

^c^ Excluded “do not know” from the trend analysis.

^d^ Participants who at baseline reported testing positive for SARS-CoV-2 were excluded from the analyses to reduce risk of reverse causation (n = 174).

**Table 3.** Sleep Score Before the COVID-19 Pandemic (2015-2017) and Sleep Dimensions Early in the COVID-19 Pandemic (April to August 2020) With Risk of PCC^a^

Sleep health		No. with PCC/total No. with COVID-19, (%)	Model adjusted for demographic and health-related factors, RR (95% CI)
Prepandemic sleep score^b^	No. of healthy pandemic sleep dimensions^c^		
Healthy (4-5)	Both (2)	190/559 (34.0)	0.64 (0.50-0.82)
	Either (1)	68/182 (37.4)	0.68 (0.50-0.93)
	Neither (0)	14/33 (42.4)	0.72 (0.41-1.27)
Intermediate or poor (0-3)	Both (2)	229/478 (47.9)	0.86 (0.68-1.08)
	Either (1)	170/372 (45.7)	0.80 (0.63-1.03)
	Neither (0)	101/180 (56.1)	1 [Reference]

Abbreviations: PCC, post–COVID-19 condition; RR, relative risk.

^a^ Participants who at baseline reported testing positive for SARS-CoV-2 were excluded from analyses to reduce risk of reverse causation (n = 174). Adjusted for age, race and ethnicity, frontline health care worker status, smoking history, body mass index, Alternate Healthy Eating Index-2010 score, alcohol intake, physical activity, and history of comorbidities.

^b^ Sleep score included morning chronotype, 7 to 8 hours per day of sleep, low insomnia symptoms, no self-reported snoring, and not at all or a little daytime dysfunction.

^c^ Sleep dimensions early in the COVID-19 pandemic included sleep duration of 7 to 8 hours per day and good or fairly good sleep quality.

Results were comparable in sensitivity analyses defining PCC as symptoms lasting 8 or more weeks; defining PCC as ongoing symptoms at the time of PCC assessment; excluding participants whose only PCC symptoms were fatigue, headache, brain fog, memory issues, and depression; including presumed COVID-19 cases without a positive test; additionally adjusting for prepandemic memory issues; excluding participants who were hospitalized due to COVID-19, vaccinated, or with probable depression and anxiety at baseline; or using multiple imputation for missing exposure and outcome data (eTables 5 and 6 in Supplement 1).

## Discussion

In this cohort study of female health care workers participating in the NHS II, we found that multiple sleep dimensions measured both before (2015-2017) and early (April to August 2020) during the pandemic were associated with a lower risk of developing PCC. A combined prepandemic sleep score comprising early chronotype, sleep duration of 7 to 8 hours per day, low insomnia symptoms, no self-reported snoring, and no frequent daytime dysfunction was inversely associated with risk of PCC. Compared with women with a 0 or 1 healthy sleep dimension, those who scored 5 had a 30% lower risk of PCC. This association was not explained by the severity of acute phase disease or depression and anxiety. In mutually adjusted models, these associations were primarily driven by daytime dysfunction. Higher self-perceived sleep quality early in the COVID-19 pandemic was also associated with a lower risk of PCC. Women with consistently healthy sleep at both times had the lowest risk of PCC compared with women with consistently unhealthy sleep.

Poorer sleep has been linked to worse COVID-19 outcomes, including hospitalization and mortality.^15^ Observations from a prospective study using data from the UK Biobank (n = 46 535) indicated that an unhealthy sleep score, including duration, daytime sleepiness, insomnia, and chronotype, measured 10 to 14 years before infection was dose-dependently associated with a higher risk of COVID-19 hospitalization and mortality^15^; persons with the least healthy sleep pattern had almost twice the mortality risk of those with the most healthy pattern. Although the association between multidimensional sleep health and PCC has not been studied, a single, large, medical record–based study of 400 000 nonhospitalized individuals with confirmed SARS-CoV-2 infection found that obstructive sleep apnea was associated with a borderline increased risk of PCC (adjusted RR, 1.07; 95% CI, 0.99-1.17).^50^ These results may underestimate this association, however, because clinically undiagnosed obstructive sleep apnea tends to be highly prevalent in the general population and is not accurately captured by medical records. The prevalence of obstructive sleep apnea was only 1.4% in this cohort^50^ compared with an estimated 10% to 15% prevalence in North America.^51,52^ Findings from a second study suggested that poor sleep quality at COVID-19 symptom onset is associated with slow recovery from COVID-19 lymphopenia.^53^

Several pathophysiologic pathways may underlie our findings. All of the dimensions of sleep we examined have been linked to systemic inflammation and immune aberrations.^54,55,56,57^ The proinflammatory state may predispose those with poor sleep health to the occurrence of cytokine storms, which have been proposed to be involved in the multiorgan manifestations of PCC.^22,24^ Furthermore, short sleep duration and insomnia have been implicated in the development of autoimmune antibodies, which have also been found in higher concentrations among patients with persistent COVID-19–related symptoms.^58,59^ Dysregulated gut microbiome, possibly through immune function, may also be a shared pathologic process between sleep disorders and PCC.^24,60^ Coagulopathy, a downstream consequence of sleep disorders, has also been observed in some patients with PCC.^61,62^

### Strengths and Limitations

A strength of this study is the longitudinal design, which allowed us to collect information on multiple sleep dimensions both before and during the pandemic. We also incorporated a multidimensional sleep score to account for the complexities of interconnected sleep behaviors and reduce measurement error. In addition, SARS-CoV-2 infection, hospitalization due to COVID-19, and vaccination against COVID-19 were prospectively collected, with frequent follow-up spanning 19 months during an active phase of the pandemic.

Our study also has several limitations. First, participants were women at middle-age or older and were relatively homogeneous in race (97% White), limiting generalizability of the findings. In addition, nearly one-half of the participants were frontline health care workers, although we did not find differences in the observed associations in frontline health care workers vs those who were not. Second, as the incidence of PCC may differ by SARS-CoV-2 strain,^6^ our findings may not apply to currently prevalent strains. Third, exposure and outcome were not missing at random, which may have introduced bias. Nevertheless, results were similar in the analysis using multiple imputation. Fourth, because SARS-CoV-2 infection and PCC were self-reported, some misclassification is likely. However, the validity of self-reported health has been high in this cohort.^63,64^ Fifth, the relatively small sample size limits our ability to detect some modest associations (eg, individuals who are long sleepers or habitual snorers).

## Conclusions

The findings from this cohort study suggest that healthy sleep before and during the COVID-19 pandemic, prior to infection, was associated with a reduced risk of PCC. Future research should investigate whether improving sleep health may prevent or alleviate PCC symptoms.

## Notes

Supplement 1.
eMethods.
**eFigure 1.** Flowchart of COVID Substudy Study Design, the Nurses’ Health Study II (NHSII), 2015-2021 **eFigure 2.** Correlation of Healthy Sleep Dimensions Prior to the COVID-19 Pandemic (2015-2017) and Early in the COVID-19 Pandemic (April-August 2020) **eTable 1.** Age-Standardized Characteristics According to Missingness of Post–COVID-19 Condition Information **eTable 2.** Age-Standardized Characteristics According to Health Care Worker Status at COVID-19 Study Baseline **eTable 3.** Sleep Score (0-5) Prior to the COVID-19 Pandemic (2015-2017) and Risk of Post–COVID-19 Condition, Stratifying by Health Care Worker Status **eTable 4.** Associations of Individual Sleep Dimensions (Dichotomized) Prior to the COVID-19 Pandemic (2015-2017) and Early in the COVID-19 Pandemic (April-August 2020) With Risk of Post–COVID-19 Condition **eTable 5.** Sensitivity Analyses of Sleep Score Prior to the COVID-19 Pandemic (2015-2017) and Risk of Post–COVID-19 Condition, Adjusting for Depression and Anxiety at COVID-19 Substudy Baseline (April-August 2020) **eTable 6.** Sensitivity Analyses of Sleep Score Prior to the COVID-19 Pandemic (2015-2017) and Risk of Post–COVID-19 Condition

Supplement 2.
Data Sharing Statement

## References

[1] Long COVID or post-COVID conditions. Centers for Disease Control and Prevention. Accessed August 24, 2022. https://www.cdc.gov/coronavirus/2019-ncov/long-term-effects/index.html

[2] Nearly one in five American adults who have had COVID-19 still have “long COVID.” Centers for Disease Control and Prevention. Accessed August 24, 2022. https://www.cdc.gov/nchs/pressroom/nchs_press_releases/2022/20220622.htm

[3] COVID-19 for health professionals: post COVID-19 condition (long COVID). Government of Canada. Accessed September 22, 2022. https://www.canada.ca/en/public-health/services/diseases/2019-novel-coronavirus-infection/health-professionals/post-covid-19-condition.html

[4] FAIR Health, Inc. *A Detailed Study of Patients With Long-Haul COVID: An Analysis of Private Healthcare Claims*. FAIR Health, Inc; 2021. Accessed June 7, 2022. https://digirepo.nlm.nih.gov/master/borndig/9918334383006676/9918334383006676.pdf

[5] Azzolini E, Levi R, Sarti R, . Association between BNT162b2 vaccination and long COVID after infections not requiring hospitalization in health care workers. JAMA. 2022;328(7):676-678. doi:10.1001/jama.2022.11691 35796131 PMC9250078

[6] Antonelli M, Pujol JC, Spector TD, Ourselin S, Steves CJ. Risk of long COVID associated with Delta versus Omicron variants of SARS-CoV-2. Lancet. 2022;399(10343):2263-2264. doi:10.1016/S0140-6736(22)00941-2 35717982 PMC9212672

[7] Nasserie T, Hittle M, Goodman SN. Assessment of the frequency and variety of persistent symptoms among patients with COVID-19: a systematic review. JAMA Netw Open. 2021;4(5):e2111417. doi:10.1001/jamanetworkopen.2021.11417 34037731 PMC8155823

[8] Groff D, Sun A, Ssentongo AE, . Short-term and long-term rates of postacute sequelae of SARS-CoV-2 infection: a systematic review. JAMA Netw Open. 2021;4(10):e2128568. doi:10.1001/jamanetworkopen.2021.28568 34643720 PMC8515212

[9] Sudre CH, Murray B, Varsavsky T, . Attributes and predictors of long COVID. Nat Med. 2021;27(4):626-631. doi:10.1038/s41591-021-01292-y 33692530 PMC7611399

[10] Wulf Hanson S, Abbafati C, Aerts JG, ; Global Burden of Disease Long COVID Collaborators. Estimated global proportions of individuals with persistent fatigue, cognitive, and respiratory symptom clusters following symptomatic COVID-19 in 2020 and 2021. JAMA. 2022;328(16):1604-1615. doi:10.1001/jama.2022.18931 36215063 PMC9552043

[11] Doi Y, Minowa M, Uchiyama M, Okawa M. Subjective sleep quality and sleep problems in the general Japanese adult population. Psychiatry Clin Neurosci. 2001;55(3):213-215. doi:10.1046/j.1440-1819.2001.00830.x 11422846

[12] Colten HR, Altevogt BM, eds. Sleep Disorders and Sleep Deprivation: An Unmet Public Health Problem. National Academies; 2006. 20669438

[13] Morin CM, Vézina-Im LA, Ivers H, . Prevalent, incident, and persistent insomnia in a population-based cohort tested before (2018) and during the first-wave of COVID-19 pandemic (2020). Sleep. 2022;45(1):zsab258. doi:10.1093/sleep/zsab258 34698868 PMC8574325

[14] Jahrami H, BaHammam AS, Bragazzi NL, Saif Z, Faris M, Vitiello MV. Sleep problems during the COVID-19 pandemic by population: a systematic review and meta-analysis. J Clin Sleep Med. 2021;17(2):299-313. doi:10.5664/jcsm.8930 33108269 PMC7853219

[15] Li P, Zheng X, Ulsa MC, . Poor sleep behavior burden and risk of COVID-19 mortality and hospitalization. Sleep. 2021;44(8):zsab138. doi:10.1093/sleep/zsab138 34142713 PMC8361340

[16] Liu Z, Luo Y, Su Y, . Associations of sleep and circadian phenotypes with COVID-19 susceptibility and hospitalization: an observational cohort study based on the UK Biobank and a two-sample Mendelian randomization study. Sleep. 2022;45(6):zsac003. doi:10.1093/sleep/zsac003 35034128 PMC8807236

[17] Peng L, Jing J, Ma J, He S, Gao X, Wang T. Insomnia and sleep duration on COVID-19 susceptibility and hospitalization: a Mendelian randomization study. Front Public Health. 2022;10:995664. doi:10.3389/fpubh.2022.995664 36249224 PMC9561394

[18] Hariyanto TI, Kurniawan A. Obstructive sleep apnea (OSA) and outcomes from coronavirus disease 2019 (COVID-19) pneumonia: a systematic review and meta-analysis. Sleep Med. 2021;82:47-53. doi:10.1016/j.sleep.2021.03.029 33892451 PMC8012298

[19] Jones SE, Maisha FI, Strausz SJ, ; FinnGen. The public health impact of poor sleep on severe COVID-19, influenza and upper respiratory infections. medRxiv. Preprint posted online February 17, 2022. doi:10.1101/2022.02.16.22271055 37301713 PMC10248098

[20] Toth LA. Sleep, sleep deprivation and infectious disease: studies in animals. Adv Neuroimmunol. 1995;5(1):79-92. doi:10.1016/0960-5428(94)00045-P 7795895

[21] Fan M, Sun D, Zhou T, . Sleep patterns, genetic susceptibility, and incident cardiovascular disease: a prospective study of 385 292 UK Biobank participants. Eur Heart J. 2020;41(11):1182-1189. doi:10.1093/eurheartj/ehz849 31848595 PMC7071844

[22] Crook H, Raza S, Nowell J, Young M, Edison P. Long COVID-mechanisms, risk factors, and management. BMJ. 2021;374(1648):n1648. doi:10.1136/bmj.n1648 34312178

[23] Behan PO, Behan WM, Gow JW, Cavanagh H, Gillespie S. Enteroviruses and postviral fatigue syndrome. Ciba Found Symp. 1993;173:146-154. doi:10.1002/9780470514382.ch9 8387908

[24] Choutka J, Jansari V, Hornig M, Iwasaki A. Unexplained post-acute infection syndromes. Nat Med. 2022;28(5):911-923. doi:10.1038/s41591-022-01810-6 35585196

[25] Besedovsky L, Lange T, Haack M. The sleep-immune crosstalk in health and disease. Physiol Rev. 2019;99(3):1325-1380. doi:10.1152/physrev.00010.2018 30920354 PMC6689741

[26] Bao Y, Bertoia ML, Lenart EB, . Origin, methods, and evolution of the Three Nurses’ Health Studies. Am J Public Health. 2016;106(9):1573-1581. doi:10.2105/AJPH.2016.303338 27459450 PMC4981810

[27] Kantermann T, Sung H, Burgess HJ. Comparing the Morningness-Eveningness Questionnaire and Munich ChronoType Questionnaire to the dim light melatonin onset. J Biol Rhythms. 2015;30(5):449-453. doi:10.1177/0748730415597520 26243627 PMC4580371

[28] Arendt J. Melatonin and human rhythms. Chronobiol Int. 2006;23(1-2):21-37. doi:10.1080/07420520500464361 16687277

[29] Patel SR, Ayas NT, Malhotra MR, . A prospective study of sleep duration and mortality risk in women. Sleep. 2004;27(3):440-444. doi:10.1093/sleep/27.3.440 15164896

[30] Levine DW, Dailey ME, Rockhill B, Tipping D, Naughton MJ, Shumaker SA. Validation of the Women’s Health Initiative Insomnia Rating Scale in a multicenter controlled clinical trial. Psychosom Med. 2005;67(1):98-104. doi:10.1097/01.psy.0000151743.58067.f0 15673630

[31] Sands-Lincoln M, Loucks EB, Lu B, . Sleep duration, insomnia, and coronary heart disease among postmenopausal women in the Women’s Health Initiative. J Womens Health (Larchmt). 2013;22(6):477-486. doi:10.1089/jwh.2012.3918 23651054 PMC3678565

[32] Bliwise DL, Nekich JC, Dement WC. Relative validity of self-reported snoring as a symptom of sleep apnea in a sleep clinic population. Chest. 1991;99(3):600-608. doi:10.1378/chest.99.3.600 1995215

[33] Harvey AG, Stinson K, Whitaker KL, Moskovitz D, Virk H. The subjective meaning of sleep quality: a comparison of individuals with and without insomnia. Sleep. 2008;31(3):383-393. doi:10.1093/sleep/31.3.383 18363315 PMC2276747

[34] Li X, Xue Q, Wang M, . Adherence to a healthy sleep pattern and incident heart failure: a prospective study of 408 802 UK Biobank participants. Circulation. 2021;143(1):97-99. doi:10.1161/CIRCULATIONAHA.120.050792 33190528 PMC7775332

[35] Geng T, Li X, Ma H, Heianza Y, Qi L. Adherence to a healthy sleep pattern and risk of chronic kidney disease: the UK Biobank study. Mayo Clin Proc. 2022;97(1):68-77. doi:10.1016/j.mayocp.2021.08.028 34996567 PMC8851869

[36] Sambou ML, Zhao X, Hong T, . Associations between sleep quality and health span: a prospective cohort study based on 328,850 UK Biobank participants. Front Genet. 2021;12:663449. doi:10.3389/fgene.2021.663449 34211497 PMC8239359

[37] Leatherby L. What previous COVID-19 waves tell us about the virus now. *New York Times*. October 23, 2021. Accessed April 11, 2023. https://www.nytimes.com/interactive/2021/10/23/us/covid-surges.html

[38] Wang S, Quan L, Chavarro JE, . Associations of depression, anxiety, worry, perceived stress, and loneliness prior to infection with risk of post-COVID-19 conditions. JAMA Psychiatry. 2022;79(11):1081-1091. doi:10.1001/jamapsychiatry.2022.2640 36069885 PMC9453634

[39] Khullar D, Zhang Y, Zang C, . Racial/ethnic disparities in post-acute sequelae of SARS-CoV-2 infection in New York: an EHR-based cohort study from the RECOVER program. J Gen Intern Med. 2023;38(5):1127-1136. doi:10.1007/s11606-022-07997-1 36795327 PMC9933823

[40] Johnson DA, Jackson CL, Williams NJ, Alcántara C. Are sleep patterns influenced by race/ethnicity—a marker of relative advantage or disadvantage? evidence to date. Nat Sci Sleep. 2019;11:79-95. doi:10.2147/NSS.S169312 31440109 PMC6664254

[41] Yuan C, Spiegelman D, Rimm EB, . Validity of a dietary questionnaire assessed by comparison with multiple weighed dietary records or 24-hour recalls. Am J Epidemiol. 2017;185(7):570-584. doi:10.1093/aje/kww104 28338828 PMC5859994

[42] Yuan C, Spiegelman D, Rimm EB, . Relative validity of nutrient intakes assessed by questionnaire, 24-hour recalls, and diet records as compared with urinary recovery and plasma concentration biomarkers: findings for women. Am J Epidemiol. 2018;187(5):1051-1063. doi:10.1093/aje/kwx328 29036411 PMC5928456

[43] Go RC, Duke LW, Harrell LE, . Development and validation of a Structured Telephone Interview for Dementia Assessment (STIDA): the NIMH Genetics Initiative. J Geriatr Psychiatry Neurol. 1997;10(4):161-167. doi:10.1177/089198879701000407 9453683

[44] Amariglio RE, Townsend MK, Grodstein F, Sperling RA, Rentz DM. Specific subjective memory complaints in older persons may indicate poor cognitive function. J Am Geriatr Soc. 2011;59(9):1612-1617. doi:10.1111/j.1532-5415.2011.03543.x 21919893 PMC3315361

[45] Löwe B, Wahl I, Rose M, . A 4-item measure of depression and anxiety: validation and standardization of the Patient Health Questionnaire-4 (PHQ-4) in the general population. J Affect Disord. 2010;122(1-2):86-95. doi:10.1016/j.jad.2009.06.019 19616305

[46] Zhou XH, Eckert GJ, Tierney WM. Multiple imputation in public health research. Stat Med. 2001;20(9-10):1541-1549. doi:10.1002/sim.689 11343373

[47] Centers for Disease Control and Prevention. How much sleep do I need? 2017. Accessed June 8, 2022. https://www.cdc.gov/sleep/about_sleep/how_much_sleep.html

[48] Qiu D, Yu Y, Li RQ, Li YL, Xiao SY. Prevalence of sleep disturbances in Chinese healthcare professionals: a systematic review and meta-analysis. Sleep Med. 2020;67:258-266. doi:10.1016/j.sleep.2019.01.047 31040078

[49] van Buuren S. Multiple imputation of discrete and continuous data by fully conditional specification. Stat Methods Med Res. 2007;16(3):219-242. doi:10.1177/0962280206074463 17621469

[50] Subramanian A, Nirantharakumar K, Hughes S, . Symptoms and risk factors for long COVID in non-hospitalized adults. Nat Med. 2022;28(8):1706-1714. doi:10.1038/s41591-022-01909-w 35879616 PMC9388369

[51] Peppard PE, Young T, Barnet JH, Palta M, Hagen EW, Hla KM. Increased prevalence of sleep-disordered breathing in adults. Am J Epidemiol. 2013;177(9):1006-1014. doi:10.1093/aje/kws342 23589584 PMC3639722

[52] Young T, Palta M, Dempsey J, Peppard PE, Nieto FJ, Hla KM. Burden of sleep apnea: rationale, design, and major findings of the Wisconsin Sleep Cohort study. WMJ. 2009;108(5):246-249. 19743755 PMC2858234

[53] Zhang J, Xu D, Xie B, . Poor-sleep is associated with slow recovery from lymphopenia and an increased need for ICU care in hospitalized patients with COVID-19: a retrospective cohort study. Brain Behav Immun. 2020;88:50-58. doi:10.1016/j.bbi.2020.05.075 32512133 PMC7274970

[54] de Punder K, Heim C, Entringer S. Association between chronotype and body mass index: the role of C-reactive protein and the cortisol response to stress. Psychoneuroendocrinology. 2019;109:104388. doi:10.1016/j.psyneuen.2019.104388 31398588

[55] Irwin MR, Olmstead R, Carroll JE. Sleep disturbance, sleep duration, and inflammation: a systematic review and meta-analysis of cohort studies and experimental sleep deprivation. Biol Psychiatry. 2016;80(1):40-52. doi:10.1016/j.biopsych.2015.05.014 26140821 PMC4666828

[56] Punjabi NM, Beamer BA. C-reactive protein is associated with sleep disordered breathing independent of adiposity. Sleep. 2007;30(1):29-34. doi:10.1093/sleep/30.1.29 17310862 PMC1978354

[57] Simpson N, Dinges DF. Sleep and inflammation. Nutr Rev. 2007;65(12, pt 2):S244-S252. doi:10.1301/nr.2007.dec.S244-S252 18240557

[58] Ni J, Zhou W, Cen H, . Evidence for causal effects of sleep disturbances on risk for osteoarthritis: a univariable and multivariable Mendelian randomization study. Osteoarthritis Cartilage. 2022;30(3):443-450. doi:10.1016/j.joca.2021.11.021 34890811

[59] Son K, Jamil R, Chowdhury A, . Circulating anti-nuclear autoantibodies in COVID-19 survivors predict long COVID symptoms. Eur Respir J. 2023;61(1):2200970. doi:10.1183/13993003.00970-2022 36137590 PMC9515477

[60] Matenchuk BA, Mandhane PJ, Kozyrskyj AL. Sleep, circadian rhythm, and gut microbiota. Sleep Med Rev. 2020;53:101340. doi:10.1016/j.smrv.2020.101340 32668369

[61] Pinotti M, Bertolucci C, Frigato E, . Chronic sleep deprivation markedly reduces coagulation factor VII expression. Haematologica. 2010;95(8):1429-1432. doi:10.3324/haematol.2010.022475 20418241 PMC2913095

[62] Prasannan N, Heightman M, Hillman T, . Impaired exercise capacity in post-COVID-19 syndrome: the role of VWF-ADAMTS13 axis. Blood Adv. 2022;6(13):4041-4048. doi:10.1182/bloodadvances.2021006944 35543533 PMC9098525

[63] Wolf AM, Hunter DJ, Colditz GA, . Reproducibility and validity of a self-administered physical activity questionnaire. Int J Epidemiol. 1994;23(5):991-999. doi:10.1093/ije/23.5.991 7860180

[64] Troy LM, Hunter DJ, Manson JE, Colditz GA, Stampfer MJ, Willett WC. The validity of recalled weight among younger women. Int J Obes Relat Metab Disord. 1995;19(8):570-572. 7489028

