# Supplementary material for: Multidimensional Sleep Health Prior to SARS-CoV-2 Infection and Risk of Post–COVID-19 Condition
Source: JAMA Netw Open. 2023 May 30;6(5):e2315885. doi: 10.1001/jamanetworkopen.2023.15885 (PMC10230315; doi:10.1001/jamanetworkopen.2023.15885)
Supplement: Supplement 1. — eMethods. eFigure 1. Flowchart of COVID Substudy Study Design, the Nurses’ Health Study II (NHSII), 2015-2021 eFigure 2. Correlation of Healthy Sleep Dimensions Prior to the COVID-19 Pandemic (2015-2017) and Early in the COVID-19 Pandemic (April-August 2020) eTable 1. Age-Standardized Characteristics According to Missingness of Post–COVID-19 Condition Information eTable 2. Age-Standardized Characteristics According to Health Care Worker Status at COVID-19 Study Baseline eTable 3. Sleep Score (0-5) Prior to the COVID-19 Pandemic (2015-2017) and Risk of Post–COVID-19 Condition, Stratifying by Health Care Worker Status eTable 4. Associations of Individual Sleep Dimensions (Dichotomized) Prior to the COVID-19 Pandemic (2015-2017) and Early in the COVID-19 Pandemic (April-August 2020) With Risk of Post–COVID-19 Condition eTable 5. Sensitivity Analyses of Sleep Score Prior to the COVID-19 Pandemic (2015-2017) and Risk of Post–COVID-19 Condition, Adjusting for Depression and Anxiety at COVID-19 Substudy Baseline (April-August 2020) eTable 6. Sensitivity Analyses of Sleep Score Prior to the COVID-19 Pandemic (2015-2017) and Risk of Post–COVID-19 Condition [file jamanetwopen-e2315885-s001.pdf]

Supplemental Online Content

Wang S, Huang T, Weisskopf MG, Kang JH, Chavarro JE, Roberts AL. Multidimensional sleep health prior to SARS-CoV-2 infection and risk of post–COVID-19 condition. *JAMA Netw Open*. 2023;6(5):e2315885. doi:10.1001/jamanetworkopen.2023.15885

eMethods.

eFigure 1. Flowchart of COVID Substudy Study Design, the Nurses' Health Study II (NHSII), 2015-2021

eFigure 2. Correlation of Healthy Sleep Dimensions Prior to the COVID-19 Pandemic (2015-2017) and Early in the COVID-19 Pandemic (April-August 2020)

eTable 1. Age-Standardized Characteristics According to Missingness of Post–COVID-19 Condition Information

eTable 2. Age-Standardized Characteristics According to Health Care Worker Status at COVID-19 Study Baseline

eTable 3. Sleep Score (0-5) Prior to the COVID-19 Pandemic (2015-2017) and Risk of Post–COVID-19 Condition, Stratifying by Health Care Worker Status

eTable 4. Associations of Individual Sleep Dimensions (Dichotomized) Prior to the COVID-19 Pandemic (2015-2017) and Early in the COVID-19 Pandemic (April-August 2020) With Risk of Post–COVID-19 Condition

eTable 5. Sensitivity Analyses of Sleep Score Prior to the COVID-19 Pandemic (2015-2017) and Risk of Post–COVID-19 Condition, Adjusting for Depression and Anxiety at COVID-19 Substudy Baseline (April-August 2020)

eTable 6. Sensitivity Analyses of Sleep Score Prior to the COVID-19 Pandemic (2015-2017) and Risk of Post–COVID-19 Condition

This supplemental material has been provided by the authors to give readers additional information about their work.

© 2023 Wang S et al. *JAMA Network Open*.

eMethods.

The Nurses' Health Study II (NHSII) was established in 1989, to study the long-term health effects of oral contraceptive use. 116,429 registered nurses of reproductive ages (25 to 42 years) residing in 1 of the 14 states (California, Connecticut, Indiana, Iowa, Kentucky, Massachusetts, Michigan, Missouri, New York, North Carolina, Ohio, Pennsylvania, South Carolina, and Texas) were enrolled. Mailed questionnaires were sent every two years, updating demographic, lifestyle, and reproductive factors, as well as medical events. Starting 2001, online versions of questionnaires were available as well.

In April 2020, a web-based COVID-19 sub-study was launched to study participants' experiences during the COVID-19 pandemic. During April and May 2020, we invited participants to join the sub-study. Exclusion criteria include: 1) died (n=5,844); 2) main cohort questionnaire not yet returned (n=21,076); 3) loss to follow-up (4,868); 4) no email address (n=26,610); 5) receiving paper questionnaire only (n=585); 6) currently in other sub-studies (n=1,104); 7) opted-out of sub-studies (n=369); 8) self-report of dementia (n=84).

© 2023 Wang S et al. *JAMA Network Open*.

eFigure 1. Flowchart of COVID Substudy Study Design, the Nurses' Health Study II (NHSII), 2015-2021

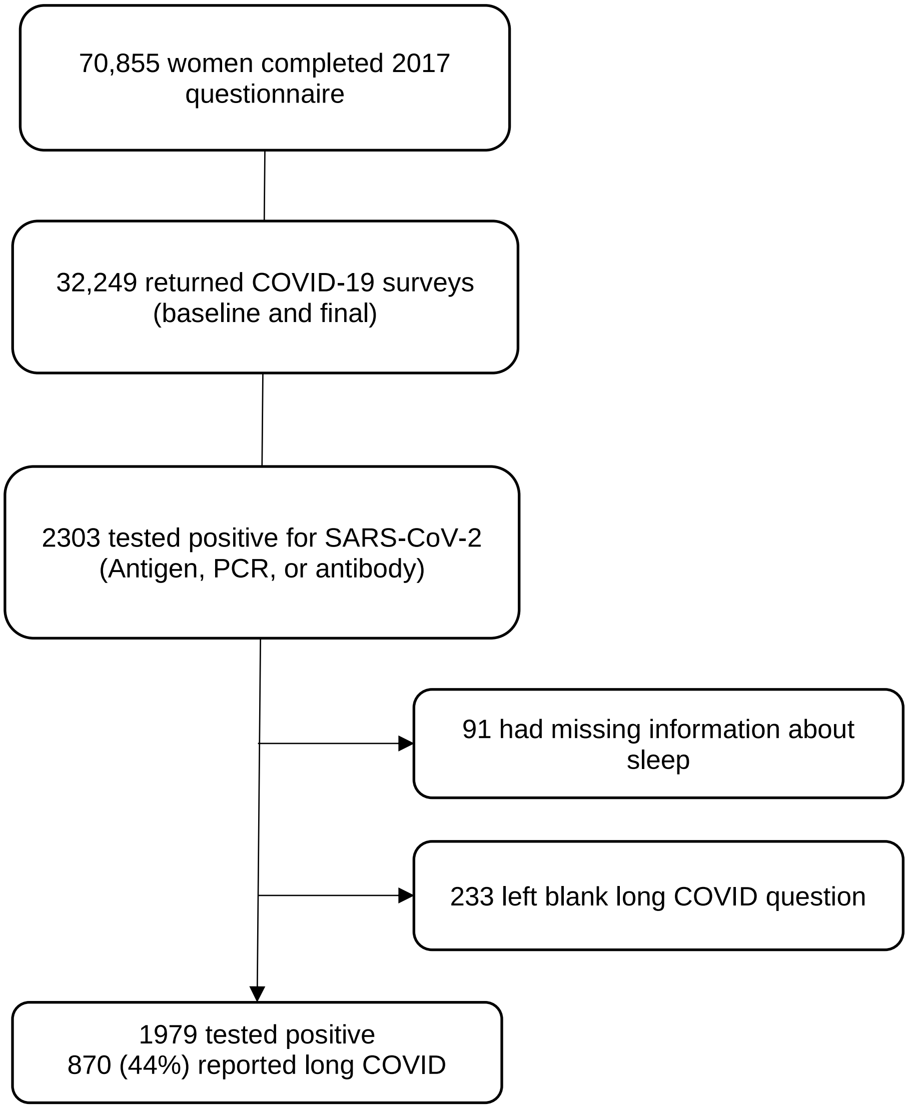

© 2023 Wang S et al. *JAMA Network Open*.

eFigure 2. Correlation of Healthy Sleep Dimensions Prior to the COVID-19 Pandemic (2015-2017) and Early in the COVID-19 Pandemic (April-August 2020))

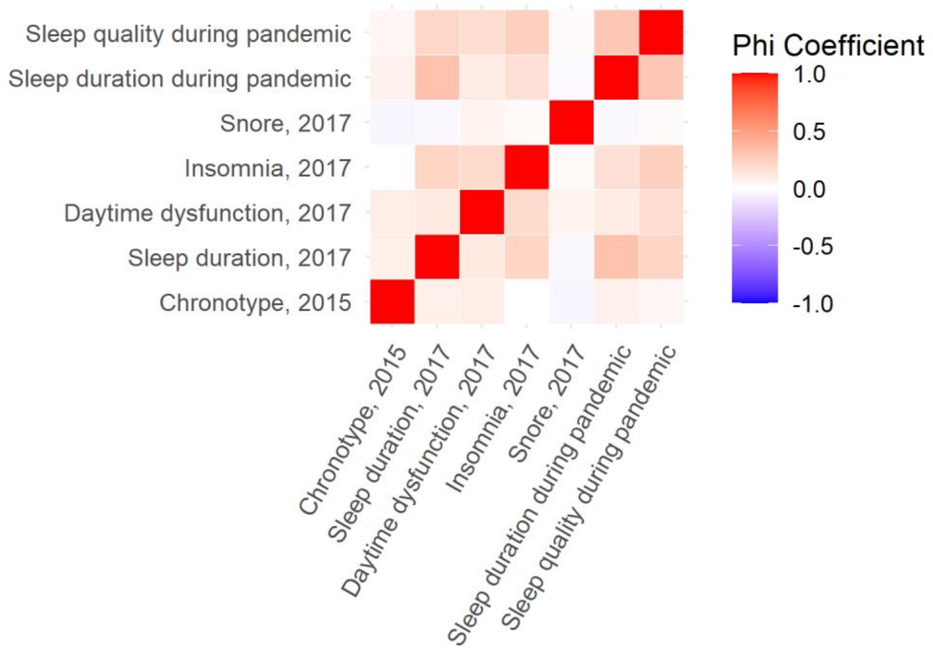

All sleep dimensions were coded as binary variables, with 1 being healthy, 0 being unhealthy: chronotype (morning, not morning), sleep duration (7–8 hours/day, <7 or >8 hours/day), insomnia symptoms (low, high), self-reported snoring (no, yes), daytime dysfunction (‘not at all’ or ‘a little’, ‘moderate amount’ or ‘quite a bit’ or ‘very much’), sleep duration during the COVID-19 pandemic (7–8 hours/day, <7 or >8 hours/day), sleep quality during the COVID-19 pandemic (‘very good’ or ‘fairly good’, ‘fairly bad’ or ‘very bad’).
Participants who at baseline reported a history of positive SARS-CoV-2 test were excluded from analyses to reduce risk of reverse causation (n=174).

© 2023 Wang S et al. *JAMA Network Open*.

eTable 1. Age-Standardized Characteristics According to Missingness of Post–COVID-19 Condition Information

	Missing exposure or outcome, n (%)	
	No<br>n=1979	Yes<br>n=324
Age, mean (SD), y[a]	64.7 (4.6)	64.6 (4.6)
Race, White	1924 (97.2)	312 (96.3)
BMI, mean (SD), kg/m^2	28.4 (6.5)	27.4 (6.4)
AHEI, mean (SD)	60.8 (12.1)	61.2 (12.3)
Alcohol, mean (SD), g/day	7.2 (11.4)	8.2 (13.4)
Physical activity, MET-hours/week	26.7 (31)	26 (29.7)
Smoking		
Never	1302 (65.8)	213 (65.8)
Past	637 (32.2)	98 (30.2)
Current	40 (2.0)	13 (4.0)
Active health worker	847 (42.8)	179 (55.2)
Depression symptoms[b], mean (SD)	0.9 (1.3)	1.0 (1.2)
Anxiety symptoms[c], mean (SD)	1.1 (1.4)	1.3 (1.4)
High cholesterol	556 (28.1)	92 (28.5)
Diabetes	159 (8.0)	17 (5.3)
Hypertension	504 (25.5)	79 (24.2)
Asthma	142 (7.2)	27 (8.3)
Cancer	131 (6.6)	29 (9.0)

[a] Not age-standardized
[b] Measured at COVID sub-study baseline (April 2020-August 2020), using the Patient Health Questionnaire 2-item (PHQ-2)
[c] Measured at COVID sub-study baseline (April 2020-August 2020), using the Generalized Anxiety Disorder 2-item (GAD-2)

© 2023 Wang S et al. *JAMA Network Open*.

eTable 2. Age-Standardized Characteristics According to Health Care Worker Status at COVID-19 Study Baseline

	Frontline healthcare worker, n (%)	
	No<br>n=1133	Yes<br>n=846
Age, mean (SD), y[a]	66.2 (4.4)	62.6 (4.1)
Race, White	1103 (97.4)	814 (96.3)
BMI, mean (SD), kg/m^2	28.5 (6.6)	28.2 (6.4)
AHEI, mean (SD)	61.1 (11.9)	60.8 (12.2)
Alcohol, mean (SD), g/day	7.1 (11.5)	8.0 (12.8)
Physical activity, MET-hours/week	26.9 (30.0)	27.6 (34.2)
Smoking		
Never	772 (68.1)	532 (62.8)
Past	337 (29.7)	297 (35.1)
Current	24 (2.1)	17 (2.0)
Depression symptoms[b], mean (SD)	0.9 (1.2)	1.0 (1.4)
Anxiety symptoms[c], mean (SD)	1.1 (1.3)	1.1 (1.4)
High cholesterol	698 (61.6)	496 (58.6)
Diabetes	144 (12.7)	88 (10.4)
Hypertension	519 (45.8)	309 (36.5)
Asthma	266 (23.4)	174 (20.6)
Cancer	258 (22.8)	165 (19.5)
Healthy sleep factors		
Morning chronotype	706 (62.3)	505 (59.7)
Sleep 7–8 hours/day	726 (64.1)	520 (61.5)
Low insomnia symptoms	750 (66.2)	580 (68.6)
No self-reported snoring	519 (45.8)	399 (47.1)
'Not at all'/ 'a little' daytime dysfunction	918 (81.1)	713 (84.3)

[a] Not age-standardized
[b] Measured at COVID sub-study baseline (April 2020-August 2020), using the Patient Health Questionnaire 2-item (PHQ-2)
[c] Measured at COVID sub-study baseline (April 2020-August 2020), using the Generalized Anxiety Disorder 2-item (GAD-2)

© 2023 Wang S et al. *JAMA Network Open*.

eTable 3. Sleep Score (0-5) Prior to the COVID-19 Pandemic (2015-2017) and Risk of Post–COVID-19 Condition, Stratifying by Health Care Worker Status

	Non-frontline healthcare worker n=1133		Frontline healthcare worker n=846	
		Main model		Main model
	Long COVID/ Total COVID	Relative Risk (95% CI)	Long COVID/ Total COVID	Relative Risk (95% CI)
Healthy sleep score				
Healthy (4–5)	57/98	1.0 [reference]	40/68	1.0 [reference]
Intermediate (2–3)	259/542	0.86 (0.64–1.15)	199/420	0.81 (0.57–1.14)
Poor (0–1)	169/493	0.66 (0.48–0.89)	146/358	0.73 (0.51–1.04)
P trend		0.002		0.09
P interaction	0.58			

Sleep score included morning chronotype, 7–8 hours/day sleep, low insomnia symptoms, no self-reported snoring, 'not at all' or 'a little' daytime dysfunction.
Relative risk and 95% confidence interval per 1 point increase in healthy sleep score, adjusting for age (continuous, years), race (White, non-White), smoking history (never, past, current), body mass index (continuous, kg/m^2), healthy eating index score (AHEI-2010, quintiles), alcohol intake (0, 0.1–4.9, 5.0–14.9, 15.0–29.9, ⩾30.0 g/day), physical activity (continuous, MET-hours/week), history of cancer (yes, no), history of diabetes (yes, no), history of asthma (yes, no), history of hypertension (yes, no), and history of high cholesterol (yes, no)

© 2023 Wang S et al. *JAMA Network Open*.

eTable 4. Associations of Individual Sleep Dimensions (Dichotomized) Prior to the COVID-19 Pandemic (2015-2017) and Early in the COVID-19 Pandemic (April-August 2020) With Risk of Post–COVID-19 Condition

Healthy sleep factors	Long COVID/ Total COVID	Adjusting for demographics, lifestyle factors, and comorbidities
		Relative Risk (95% CI)
**Prior to the pandemic**		
Chronotype		
Evening or intermediate type[a]	365/768	1.0 [reference]
Morning chronotype[b]	505/1211	0.91 (0.80–1.05)
Sleep duration, hours/day		
<7, or >8	365/735	1.0 [reference]
7–8	505/1244	0.85 (0.74–0.97)
Insomnia symptoms		
High (6–9)	316/656	1.0 [reference]
Low (0–5)	554/1323	0.87 (0.75–1.00)
Self-reported snoring		
Yes	487/1062	1.0 [reference]
No	383/917	0.94 (0.82–1.08)
Daytime dysfunction		
'Moderate'/ 'Quite a bit'/ 'Very much'	194/358	1.0 [reference]
'Not at all'/ 'a little'	676/1621	0.80 (0.68–0.94)
**Early in the pandemic**		
Sleep duration, hours/day		
<7, or >8	303/661	1.0 [reference]
7–8	469/1143	0.93 (0.80–1.08)
Sleep quality		
'fairly bad' or 'bad'	165/319	1.0 [reference]
'good' or 'fairly good'	607/1485	0.82 (0.69–0.97)

Adjusted for age (continuous, years), race (White, non-White), frontline healthcare worker (yes, no), smoking history (never, past, current), body mass index (continuous, kg/m^2), healthy eating index score (AHEI-2010, quintiles), alcohol intake (0, 0.1–4.9, 5.0–14.9, 15.0–29.9, ⩾30.0 g/day), physical activity (continuous, MET-hours/week), history of cancer (yes, no), history of diabetes (yes, no), history of asthma (yes, no), history of hypertension (yes, no), and history of high cholesterol (yes, no).
[a] included more of an evening person, definite evening type, and neither morning nor evening type
[b] included more of a morning person and definite morning type
Participants who at baseline reported a history of positive SARS-CoV-2 test were excluded from analyses to reduce risk of reverse causation (n=174).

© 2023 Wang S et al. *JAMA Network Open*.

eTable 5. Sensitivity Analyses of Sleep Score Prior to the COVID-19 Pandemic (2015-2017) and Risk of Post–COVID-19 Condition, Adjusting for Depression and Anxiety at COVID-19 Substudy Baseline (April-August 2020)

	Main model	Additionally excluded 336 persons with probable depression and anxiety at COVID-19 sub-study baseline[a]
	RR (95% CI)	
Healthy sleep score		
Healthy (4–5)	1.0 [reference]	1.0 [reference]
Intermediate (2–3)	0.83 (0.66–1.05)	0.85 (0.64–1.13)
Poor sleep (0–1)	0.65 (0.51–0.83)	0.69 (0.51–0.93)
P trend	<0.001	0.004

Sleep score included morning chronotype, 7–8 hours/day sleep, low insomnia symptoms, no self-reported snoring, ‘not at all’ or ‘a little’ daytime dysfunction.

Participants who at baseline reported a history of positive SARS-CoV-2 test were excluded from analyses to reduce risk of reverse causation (n=174).

Adjusted for age (continuous, years), race (White, non-White), frontline healthcare worker (yes, no), smoking history (never, past, current), body mass index (continuous, kg/m^2), healthy eating index score (AHEI-2010, quintiles), alcohol intake (0, 0.1–4.9, 5.0–14.9, 15.0–29.9, ≥30.0 g/day), physical activity (continuous, MET-hours/week), history of cancer (yes, no), history of diabetes (yes, no), history of asthma (yes, no), history of hypertension (yes, no), and history of high cholesterol (yes, no)

[a] Depression was measured using the Patient Health Questionnaire 2-item (PHQ-2) and anxiety was measured using the Generalized Anxiety Disorder 2-item (GAD-2)

© 2023 Wang S et al. *JAMA Network Open*.

eTable 6. Sensitivity Analyses of Sleep Score Prior to the COVID-19 Pandemic (2015-2017) and Risk of Post–COVID-19 Condition

	Main model	Defining long COVID as having ≥8 weeks of symptoms[a]	Defining long COVID as having ongoing symptoms	Excluding 155 persons reporting only fatigue, headache, brain fog, memory issue, and depression	Including 2891 self-presumed COVID-19 cases regardless of a positive test
	Relative Risk (95% CI)				
Healthy sleep score					
Healthy (4–5)	1.0 [reference]	1.0 [reference]	1.0 [reference]	1.0 [reference]	1.0 [reference]
Intermediate (2–3)	0.84 (0.67–1.05)	0.82 (0.65–1.04)	0.79 (0.62–1.02)	0.84 (0.66–1.08)	0.88 (0.73–1.06)
Poor sleep (0–1)	0.68 (0.54–0.86)	0.68 (0.53–0.87)	0.62 (0.48–0.81)	0.64 (0.50–0.84)	0.72 (0.59–0.88)
P trend	<0.001	<0.001	<0.001	<0.001	<0.001

(continued)

	Main model	Multiple imputation of missing sleep and/or long COVID information[a]	Additionally adjusted for memory issues in 2017	Excluded 102 persons who were hospitalized due to COVID-19	Excluded 129 persons who had been vaccinated at the time of SARS-CoV-2 infection
	Relative Risk (95% CI)				
Healthy sleep score					
Healthy (4–5)	1.0 [reference]	1.0 [reference]	1.0 [reference]	1.0 [reference]	1.0 [reference]
Intermediate (2–3)	0.84 (0.67–1.05)	0.84 (0.68–1.04)	0.84 (0.68–1.05)	0.82 (0.65–1.04)	0.84 (0.67–1.05)
Poor sleep (0–1)	0.68 (0.54–0.86)	0.68 (0.55–0.85)	0.69 (0.54–0.87)	0.65 (0.51–0.82)	0.68 (0.54–0.87)
P trend	<0.001	<0.001	<0.001	<0.001	<0.001

Sleep score included morning chronotype, 7–8 hours/day sleep, low insomnia symptoms, no self-reported snoring, 'not at all' or 'a little' daytime dysfunction.
Adjusted for age (continuous, years), race (White, non-White), frontline healthcare worker (yes, no), smoking history (never, past, current), body mass index (continuous, kg/m^2), healthy eating index score (AHEI-2010, quintiles), alcohol intake (0, 0.1–4.9, 5.0–14.9, 15.0–29.9, ⩾30.0 g/day), physical activity (continuous, MET-hours/week), history of cancer (yes, no), history of diabetes (yes, no), history of asthma (yes, no), history of hypertension (yes, no), and history of high cholesterol (yes, no)
[a] Participants who had an initial infection within 2 months of the long COVID assessment were excluded from the analysis (n=33).
[b] Multiple imputation was performed with fully conditional specification using 20 imputed datasets

© 2023 Wang S et al. *JAMA Network Open*.
