# Supplementary material for: Multidimensional Sleep Health Prior to SARS-CoV-2 Infection and Risk of Post–COVID-19 Condition
Source: JAMA Netw Open. 2023 May 30;6(5):e2315885. doi: 10.1001/jamanetworkopen.2023.15885 (PMC10230315; doi:10.1001/jamanetworkopen.2023.15885)
Supplement: Supplement 2. — Data Sharing Statement [file jamanetwopen-e2315885-s002.pdf]

Data Sharing Statement

Wang. Multidimensional Sleep Health Prior to SARS-CoV-2 Infection and Risk of Post–COVID-19 Condition. *JAMA Netw Open*. Published May 30, 2023.
doi:10.1001/jamanetworkopen.2023.15885

Data
Data available: No

Additional Information
Explanation for why data not available: Further information including the procedures to obtain and access data from the Nurses' Health Studies is described at https://www.nurseshealthstudy.org/researchers (contact email: nhsaccess@channing.harvard.edu).
